# Editorial: Updates in drug reaction with eosinophilia and systemic symptoms (DRESS)

**DOI:** 10.3389/fmed.2023.1305765

**Published:** 2023-10-24

**Authors:** Baptiste Janela, Yen Loo Lim, Saeko Nakajima

**Affiliations:** ^1^Lee Kong Chian School of Medicine, Nanyang Technological University, Singapore, Singapore; ^2^National Skin Centre, Singapore, Singapore; ^3^Faculty of Medicine, Kyoto University, Kyoto, Japan

**Keywords:** severe cutaneous adverse drug reaction (sCADR), drug reaction with eosinophilia and systemic symptoms (DRESS), drug induced hypersensitivity syndrome (DIHS), drug eruption, review

Five articles are published in this Frontiers' Research Topic on *Drug reaction with eosinophilia and systemic symptoms (DRESS)*.

Of these, the highlight would be the article by Chen et al., offering an elegant review of DRESS. This comprehensive review delves into the pathogenesis of DRESS, potential biomarkers, and the relevant therapeutic rationales. With our current understanding of the genetic susceptibility, models of antigen presentation, and T-cell activation by drugs, DRESS along with other severe cutaneous adverse drug reactions can no longer be dismissed as unpredictable, at least for specific drugs. Prevention of DRESS associated with some medications is possible; the most successful example is that of HLA B1502 screening prior to carbamazepine use in the Han Chinese population (1). Traditionally, the treatment of DRESS has centered on systemic corticosteroids. However, recent insights into the cytokines and chemokines involved in DRESS have opened up the possibility of more targeted treatments. However, these alternative options, such as IL-5/IL-5Receptor blockade and pan-JAK inhibitors, are still in their early stages of development and testing.

Ciclosporin, on the other hand, which is widely used for various other inflammatory dermatoses, has been demonstrated to be an effective and safe alternative treatment for recalcitrant corticosteroid-dependent DRESS or in patients where corticosteroids are contraindicated. In fact, Verstegen et al. have advocated ciclosporin as a first-line treatment based on its mechanism of action and their observations of quicker improvement and better tolerability compared to corticosteroids in DRESS.

Research in DRESS remains challenging due to its rarity, particularly when studying it within the subpopulation of people living with HIV (PLHIV). PLHIV experience a higher frequency of drug eruptions when compared with the non-HIV population (2). Immune dysregulation, polypharmacy, more frequent use of “higher-risk” drugs, and repetitive courses of anti-microbials are contributory factors to this observation. Despite the challenges due to heterogeneity of these factors, Chimbetete et al. have contributed to the literature by shedding light on the cutaneous T-cell profile of DRESS in PLHIV. While their study has its limitations, they have found that dermal FOXp3 ^+^ T cells were more frequently increased in HIV-positive DRESS and patients reacting to more than one drug. The significance of this remains to be determined and, hopefully, more studies can be done to verify the observations.

Furthermore, DRESS do occur in children, as highlighted in the case report by Kuchinskaya et al.. This report underscores the challenges in recognizing and differentiating DRESS from other systemic inflammatory syndromes with similar presentations and laboratory findings. Lastly, Manieri et al. provided us a review of DRESS in childhood and highlighted that rapid-onset DRESS affects children more often than adults and is usually triggered by antibiotics or iodinated contrast media rather than by anticonvulsants.

In conclusion, this Frontiers Research Topic provides valuable insights into the complex realm of DRESS, offering a deeper understanding of its mechanisms, treatment options, and challenges in various populations.

Enjoy the wealth of knowledge shared in this Research Topic on DRESS!

## Author contributions

BJ: Writing — original draft, Writing — review & editing. YL: Writing — original draft, Writing — review & editing. SN: Writing — original draft, Writing — review & editing.
